# KLHL18 inhibits the proliferation, migration, and invasion of non-small cell lung cancer by inhibiting PI3K/PD-L1 axis activity

**DOI:** 10.1186/s13578-020-00499-9

**Published:** 2020-11-27

**Authors:** Xizi Jiang, Yitong XU, Hongjiu Ren, Jun Jiang, Muli Wudu, Qiongzi Wang, Jingqian Guan, Hongbo Su, Yao Zhang, Bo Zhang, Yuanzi Guo, Yujiao Hu, Lihong Jiang, Zongang Liu, Huanxi Wang, Yu Cheng, Limei Sun, Xueshan Qiu

**Affiliations:** 1 Department of Pathology, College of Basic Medical Sciences, China Medical University, Shenyang, China; 2 Department of Pathology, The First Bethune Hospital of Jilin University, Changchun, Jilin China; 3 Department of Pathology, Basic Medical Sciences, Xinjiang Medical University, Urumqi, China; 4 Department of Pathology, Chang’an District Hospital, The First Affiliated Hospital of Xi’an Jiaotong University, No. 120 Wenyuan Middle Road, Guodu Street, Chang’an District, Xi’an, 710100 Shaanxi China; 5 Shengjing Hospital Affiliated With China Medical University, 19F, Building No. 1B, No. 36, Sanhao Street, Heping District, Shenyang, 110000 Liaoning China; 6 Department of Pathology, The First Hospital of China Medical University, No. 155 NanjingBei Street, Heping District, Shenyang, 110001 Liaoning People’s Republic of China

**Keywords:** Non-small cell lung cancer, Kelch-like protein 18, Diagnosis, PD-L1 protein, Ubiquitination, PI3K/AKT/mTOR pathway

## Abstract

**Background:**

The expression of Kelch-like protein 18 (KLHL18) in non-small cell lung cancer (NSCLC) is lower than that in normal lung tissue according to the Gene Expression Profiling Interactive Analysis database. KLHL18 is a BTB domain protein and binds cullin 3 (CUL3). However, whether this complex participates in ubiquitination-mediated protein degradation in NSCLC is unclear. Therefore, we aimed to investigate the role of KLHL18 in human NSCLC cells.

**Results:**

We found that KLHL18 is downregulated in cancer cells and is associated with poor prognosis. Further, its expression was significantly associated with tumor node metastasis (TNM) stage, lymph node metastasis, and tumor size. In vitro analysis of NSCLC cells showed that overexpressing KLHL18 inhibited cell proliferation, migration, and invasion. We found that the tumor-inhibitory effect of the KLHL18 protein was achieved by promoting the ubiquitination and degradation of phosphatidylinositol 3-kinase (PI3K) p85α and inhibiting the expression of PD-L1 protein, ultimately preventing tumor cell immune escape.

**Conclusions:**

Our results identified the tumor-suppressive mechanism of KLHL18 and suggested that it is closely related to NSCLC occurrence and development. Further investigation of the underlying mechanism may provide new targets for NSCLC treatment.

## Background

The incidence of lung cancer in China is increasing, leading to an enormous social and economic burden [1]. In the past few decades, non-small cell lung cancer (NSCLC) and small cell lung cancer have emerged as the most commonly used diagnostic terms for lung cancer [2–5]. In recent years, several oncogenic genes have been discovered, and studies on the effects of EGFR mutations, ALK fusions, and inhibition of hTERT overexpression suggested that these are primary therapeutic targets for NSCLC [6–8]. However, the molecular mechanisms underlying lung tumorigenesis remain unclear, making it vital to identify new therapeutic targets to improve treatment strategies for patients with lung cancer.

The phosphatidylinositol 3-kinase (PI3K)/AKT/mTOR pathway is thought to be important in carcinogenesis and plays a crucial role in many human tumors [9]. The PI3K/AKT/mTOR pathway can also increase the expression of PD-L1 at the protein level by inhibiting autophagy, thereby contributing to the tumor’s immune microenvironment [10]. In the past few years, therapeutics targeting PI3K or AKT have been widely used in clinical trials [11–14]. However, similar to other targeted therapies, adaptability and resistance limit the anti-tumor effects of these drugs. Therefore, it is particularly important to fully unveil and exploit the intra-pathway interactions for clinical treatment [15].

Cullin 3 (CUL3) can target proteins via the BTB domain and ubiquitinate its substrates [16, 17]. Kelch-like protein 18 (KLHL18) contains a BTB/POZ domain, a BACK domain, and six Kelch repeats [18]; each of these domains have multiple functions [16, 19–24]. Cullin-RING ligase (CRL) is a multi-subunit E3 ubiquitin ligase that recruits substrate-specific adaptors to catalyze protein ubiquitination [25]. Furthermore, KLHL18 reportedly interacts with CUL3 to promote their ubiquitination-mediated degradation [26]. A recent study showed that the KLHL18-CUL3 complex could degrade the UNC119 protein. Of note, KLHL18 is expressed specifically in photoreceptors in the mouse retina and was not detected in the mouse lung or other tissues [27]. However, according to the Gene Expression Profiling Interactive Analysis (GEPIA) database (http://gepia.cancer-pku.cn/), *KLHL18* is a tumor suppressor gene in NSCLC. The purpose of this study was to determine the role of KLHL18 in human NSCLC cells.

## Results

### Low expression of KLHL18 in human NSCLC is associated with poor prognosis

**Fig. 1:**
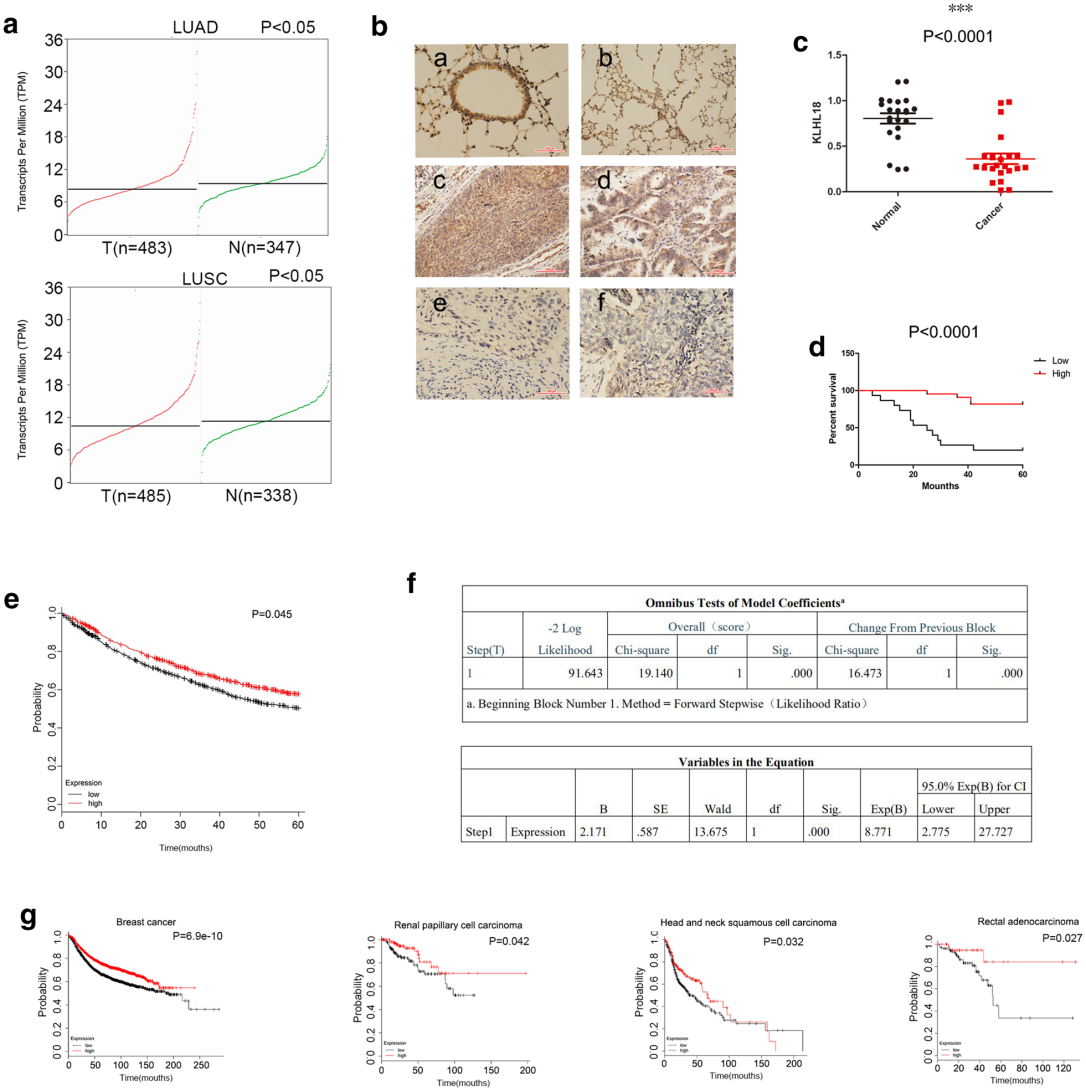
Low KLHL18 expression in human non-small cell lung cancer (NSCLC) is associated with poor prognosis. **a** KLHL18 expression in lung adenocarcinoma (LUAD) is lower than in normal lung tissue. *P* < 0.05. KLHL18 expression in lung squamous cell carcinoma (LUSC) is lower than in normal lung tissue. *P* < 0.05. **b** The expression of KLHL18 protein in immunohistochemical sections of [a] normal bronchial epithelial tissue, [b] normal alveolar tissue, [c] highly differentiated squamous cell carcinoma, [d] highly differentiated LUAD, [e] poorly differentiated squamous cell carcinoma, and [f] poorly differentiated LUAD. **c** qRT-PCR analysis of the expression level of *KLHL18* in 22 pairs of NSCLC and adjacent tissues, ***P < 0.0001. **d** Prognostic analysis of immunohistochemistry results in 37 clinical samples shows that patients with high KLHL18 protein expression had longer survival times and better prognosis. *P* < 0.05. **e** Kaplan–Meier curves showing that low-level KLHL18 protein expression was associated with poor prognosis of NSCLC, *P* = 0.0045. **f** Cox regression analysis shows that KLHL18 is an independent risk factor affecting NSCLC patients’ prognosis, *P* < 0.0001. **g** Low expression of KLHL18 protein was also associated with poor prognosis in patients with breast cancer, renal papillary cell carcinoma, head, and neck squamous cell carcinoma, and rectal adenocarcinoma, *P* < 0.05

In the GEPIA database, the gene *KLHL18* was found to have lower expression in NSCLC than in normal lung tissue (Fig. 1a; *P* < 0.05). To determine the role of KLHL18 in lung cancer, we performed immunohistochemical analysis of 214 NSCLC samples. Tumors from 128 subjects with high KLHL18 expression were highly differentiated, whereas 86 tumors that showed weak or low expression of KLHL18 were less differentiated. KLHL18 expression was inversely correlated with lymph node metastasis (*P* < 0.0001), tumor size (*P* = 0.037), and TNM stage (*P* = 0.019) (Table 1). KLHL18 was found to localize to the cytoplasm and nucleus of both bronchial epithelial cells and tumors. However, it showed strong positive expression in normal bronchial epithelial cells and weak expression in tumor cells (Fig. 1b). Next, we examined 22 pairs of NSCLC and adjacent tissues using quantitative polymerase chain reaction (qPCR) and found that the expression of *KLHL18* in adjacent tissues was significantly higher than that in cancer tissues (Fig. 1c; *P < 0.05, **P < 0.01). We selected 37 clinical samples and performed immunohistochemistry experiments to evaluate clinical prognosis. It was found that patients with high KLHL18 protein expression had good clinical prognoses (Fig. 1d; *P* < 0.0001). Kaplan–Meier survival analysis showed a significant association between low levels of KLHL18 protein and poor prognosis (Fig. 1e; *P* < 0.05). Moreover, Cox regression analysis shows that KLHL18 is an independent risk factor affecting the prognosis of NSCLC patients (Fig. 1f; *P* < 0.0001). KLHL18 expression was also correlated with a tumor suppressor function in various cancers such as breast cancer (Fig. 1g), supporting the hypothesis that *KLHL18* acts as a tumor suppressor gene.

**Table 1:** Distribution of KLHL18 status in non-small cell lung cancer according to clinicopathological characteristics

Characteristics	Number of patients	KLHL18 overexpression	KLHL18 negative or weak expression	*P*-value
Age				
≥ 50 years	139	79 (56.83%)	60 (43.17%)	0.245
< 50 years	75	49 (65.33%)	26 (34.67%)	
Sex				
Male	110	65 (59.10%)	45 (40.90%)	0.889
Female	104	63 (60.58%)	41 (39.42%)	
Histology				
Adenocarcinoma	113	68 (60.18%)	45 (39.82%)	1.000
Squamous cell carcinoma	101	60 (59.41%)	41 (40.59%)	
TNM stage				
I–II	138	91 (65.94%)	47 (34.06%)	0.019
III	76	37 (48.68%)	39 (51.32%)	
Nodal status				
N0	137	98 (71.53%)	39 (28.47%)	< 0.0001
N1, N2, N3	77	30 (38.96%)	47 (61.04%)	
Differentiation				
Well-moderate	118	68 (57.63%)	50 (42.37%)	0.487
Poor	96	60 (62.50%)	36 (37.50%)	
Tumor size				
≥ 3 cm	126	68 (53.97%)	58 (46.03%)	0.037
< 3 cm	88	60 (68.18%)	28 (31.82%)	

### Silencing KLHL18 promotes proliferation, migration, and invasion of NSCLC cells

**Fig. 2:**
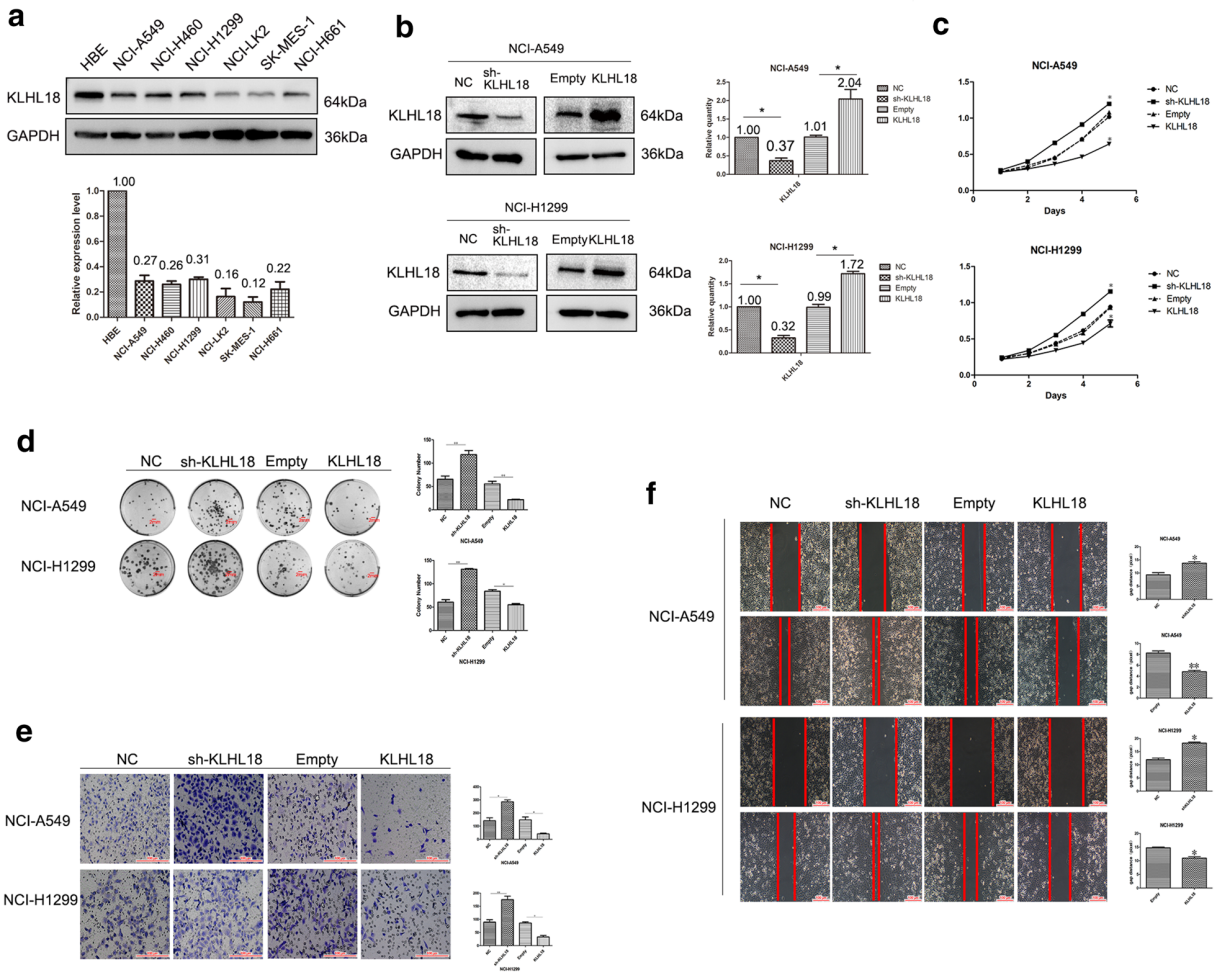
Silencing *KLHL18* promotes proliferation, migration, and invasion of non-small cell lung cancer (NSCLC) cells. **a** Proteins were extracted from one bronchial epithelial and six NSCLC cell lines to determine the KLHL18 expression level. The lower panel shows the statistical analysis of KLHL18 expression in various cell lines. **b** The knockdown and overexpression efficiency of KLHL18 in lung cancer NCI-A549 cells and NCI-H1299 cells. The histogram on the right is a statistical graph of its gray value, *P < 0.05, **P < 0.01. **c** MTS assay results of NCI-A549 cells transfected with KLHL18-shRNA and KLHL18-Flag plasmids, *P < 0.05, **P < 0.01. The upper graph is the MTS result of NCI-A549, the lower graph is the MTS result of NCI-H1299. **d** The top panel shows a colony formation assay using NCI-A549 cells transfected with KLHL18-shRNA and KLHL18-Flag plasmids, *P < 0.05, **P < 0.01, and the lower panel shows the same results of NCI-H1299 cells, *P < 0.05, **P < 0.01. The far-right panel shows a quantitative representation of the colony-forming ability of both cell lines. **e** Transwell assays using KLHL18-expressing NCI-A549 and NCI-H1299 cell lines. The right-most panel shows KLHL18 in both cell lines. The graph shows a representation of the number of cells passing through the Matrigel, *P < 0.05, **P < 0.01. **f** Wound-healing assay showing the KLHL18 effect on the NCI-A549 and NCI-H1299 cell migration. The right panel shows the migration distance of the cancer cells, *P < 0.05, **P < 0.01

Based on our findings, we selected seven common lung tissue cell lines (HBE, NCI-A549, NCI-H460, NCI-H1299, NCI-LK2, SK-MES-1, and NCI-H661 cells) to extract total protein and perform western blot analysis. KLHL18 was highly expressed in the normal bronchial epithelial cell line HBE, whereas its expression was relatively low in the other six lung cancer cell lines (Fig. 2a). Based on these results, we selected NCI-A549 and NCI-H1299 cells, with relatively moderate expression of KLHL18, as representative cancer cell lines for an in-depth study of NSCLC.

We used KLHL18-shRNA and KLHL18-Flag plasmids to alter the expression of *KLHL18* in the cell lines (Fig. 2b) and subsequently analyzed the behavioral changes in the selected cells. A reduction in *KLHL18* promoted the proliferation of NSCLC cells in vitro (Fig. 2c, d), whereas their proliferative capacity was diminished in KLHL18-Flag stably transfected cell lines. In the invasion experiment, we found that after KLHL18-shRNA transfection, the number of cells passing through the Transwell chamber was higher than that in the control group, and this cell invasion ability of the KLHL18-enhanced cell line was significantly reduced (Fig. 2e). Consistent with these observations, based on the scratch test, the migrative ability of *KLHL18*-suppressed cells was enhanced, whereas it was decreased in *KLHL18*-overexpressing cells (Fig. 2f). These results were validated in both the NCI-A549 and NCI-H1299 cell lines. Together, these results demonstrate that KLHL18 markedly inhibits the proliferation, migration, and invasion of NSCLC cells. These findings are also consistent with the previously mentioned immunohistochemical and qPCR results obtained with clinical samples, in which KLHL18 expression was associated with a decrease in tumor size, migration, and invasion.

### KLHL18 regulates the function of NSCLC cells through the PI3K-PD-L1 axis

**Fig. 3:**
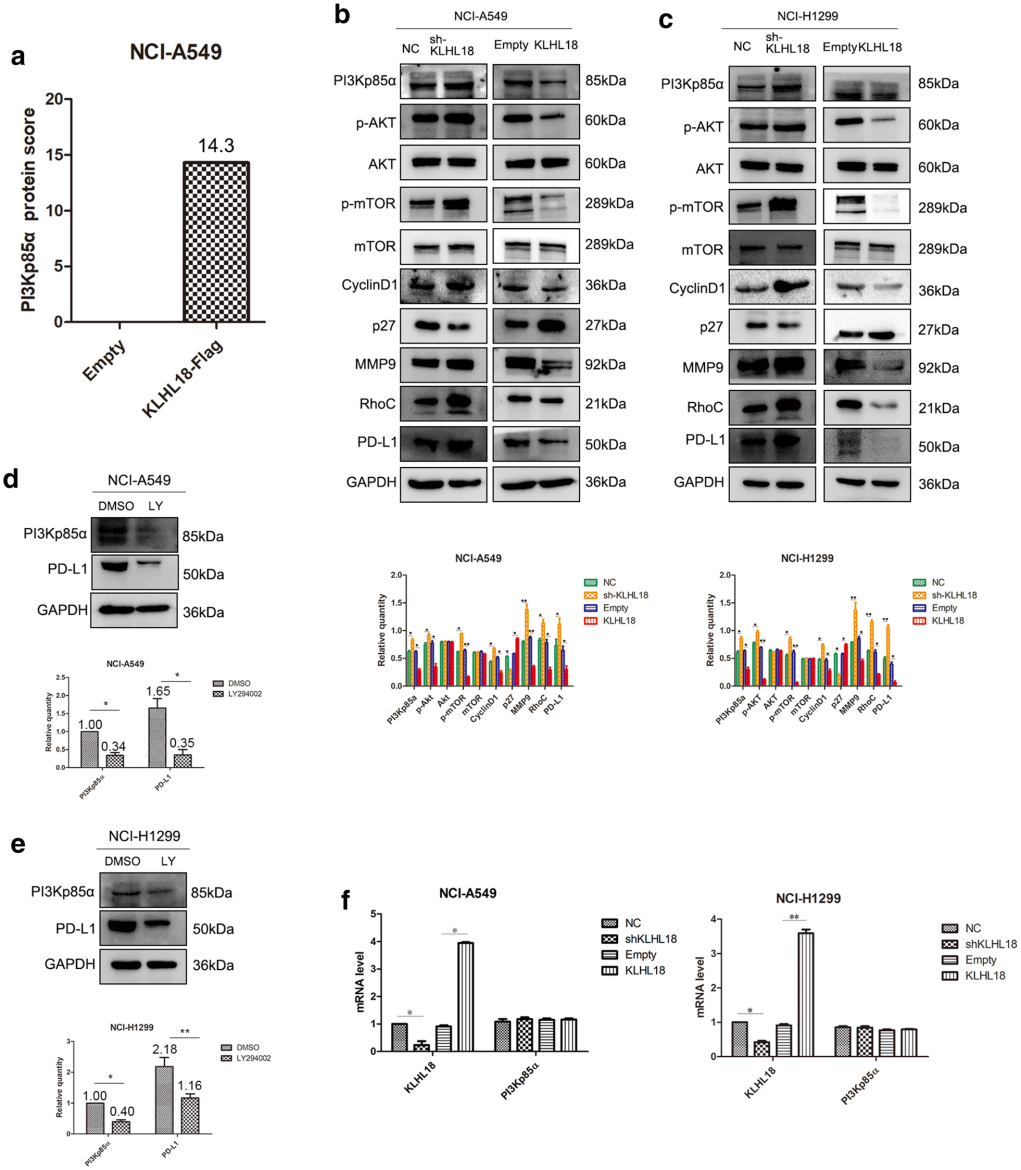
KLHL18 regulates non-small cell lung cancer cell proliferation, migration, and invasion through the PI3K/AKT/mTOR pathway. **a** Spectrometry analysis with NCI-A549 cells revealed that KLHL18 protein can bind to PI3KP85α. **b**, **c** The effect of KLHL18 protein expression on the expression of effector proteins and pathway proteins in the **b** NCI-A549 and **c** NCI-H1299 cell lines. The lower graph corresponds to the gray value statistical graph, *P < 0.05, **P < 0.01. **d**, **e** PD-L1 protein levels decreased after a PI3K pathway inhibitor was added in both NCI-A549 (**d**) and NCI-H1299 (**e**) cell lines. The lower graph corresponds to the gray value statistical graph of various protein changes, *P < 0.05, **P < 0.01. **f** In the qRT-PCR experiments, the mRNA level of *PI3KP85α* did not change with the expression of KLHL18

First, we performed mass spectrometric analysis and found that KLHL18 protein can bind to PI3Kp85α protein (Fig. 3a and Additional file 1: Fig. S1), then KLHL18 was found to modulate the activity of the PI3K/AKT/mTOR pathway. The expression of PI3Kp85α protein was decreased and the EMT pathway was inhibited in NCI-A549 cells stably transfected with KLHL18-Flag (Fig. 3b and Additional file 1: Fig. S2). The protein levels of PD-L1, related to tumor immunity, were also altered. The opposite results were obtained with NCI-A549 cells stably transfected with shKLHL18-GFP. The same result was obtained using NCI-H1299 cells (Fig. 3c and Additional file 1: Fig. S2). Taken together, these results indicate that KLHL18 inhibits the proliferation, migration, and invasion of NSCLC cells by inhibiting the activity of the PI3K/AKT pathway. In our experiments, the relationship among PD-L1, PI3K, AKT, and mTOR was confirmed. First, adding the PI3K pathway inhibitor LY294002, phosphorylation levels of AKT and mTOR decreased, and the protein levels of PD-L1 decreased. Second, after adding the mTOR inhibitor rapamycin, PD-L1 levels also decreased, but the protein levels of PI3Kp85α and the phosphorylation levels of AKT did not change (Fig. 3d, e, and Additional file 1: Fig. S3). These data indicate that the PI3K/AKT signaling pathway affects PD-L1 protein levels through mTOR. Moreover, our findings are consistent with previous reports demonstrating that the PI3K/AKT/mTOR signaling pathway can affect the expression levels of PD-L1 protein [10].

To test whether KLHL18 affects PI3Kp85α expression at the gene level, we performed qPCR. The mRNA level of *PI3Kp85α* did not change significantly, regardless of whether *KLHL18* was increased or decreased (Fig. 3f), indicating that KLHL18 did not affect the transcription level of *PI3Kp85α*.

### KLHL18 interacts with and promotes the ubiquitination and degradation of PI3Kp85α

**Fig. 4:**
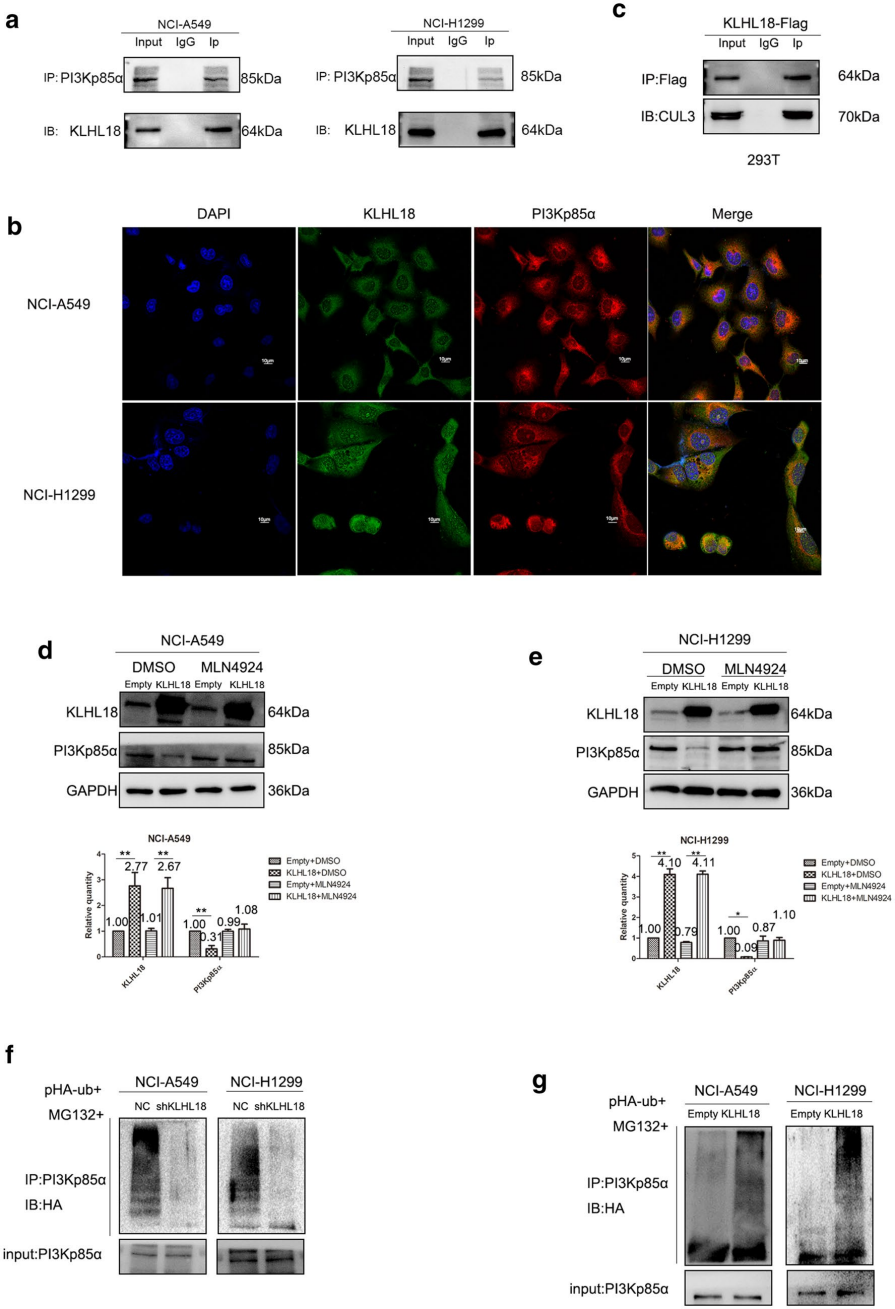
KLHL18 interacts with and promotes the ubiquitination and degradation of PI3Kp85α. **a** Co-IP of KLHL18 with PI3KP85α in NCI-A549 and NCI-H1299 cells. **b** Immunofluorescence assay; KLHL18 co-localized with PI3KP85α in NCI-A549 and NCI-H1299 cells. **c** KLHL18 protein co-immunoprecipitated with CUL3 protein in HEK-293 T cells. **d**, **e** KLHL18 inhibited the expression of PI3Kp85α via the Rbx1–CUL3–KLHL18 complex in **d** NCI-A549 and (E) NCI-H1299 cell lines. The lower graph corresponds to the gray value statistical graph, *P < 0.05, **P < 0.01. **f** Silencing KLHL18 protein inhibited the ubiquitination of PI3Kp85α protein. **g** An increase in KLHL18 protein expression levels promoted the ubiquitination-mediated degradation of PI3Kp85α protein

Co-immunoprecipitation (Co-IP) experiments revealed that KLHL18 interacts with PI3Kp85α in both NCI-A549 and NCI-H1299 cells (Fig. 4a). This result was verified by immune-co-localization experiments in both cell lines (Fig. 4b). Previous studies described that KLHL18 co-immunoprecipitates with CUL3, which, after repeated trials, could be replicated in our study (Fig. 4c); the latter is known to function in a complex with E3 ubiquitin ligase in the ubiquitin–proteasome system [17].

To demonstrate the CRL complex-dependent downregulation of PI3Kp85α protein, we pretreated NCI-A549 and NCI-H1299 cells with MLN4924, an inhibitor that blocks cullin neddylation. The results obtained after treatment with MLN4924 indicated that downregulation of PI3Kp85α requires the involvement of a functional CRL complex (Fig. 4d, e). Upon transfection of the Ub-HA plasmid into NCI-A549 and NCI-H1299 cells stably transfected with shKLHL18-GFP, the ubiquitination level of PI3Kp85α was found to be decreased after adding the proteasome inhibitor MG-132 (Fig. 4f). The same experiment was performed in both cell lines stably transfected with KLHL18-Flag and an increase in PI3Kp85α ubiquitination level was observed (Fig. 4g). These results suggest an interaction between KLHL18 and PI3Kp85α and further demonstrate that KLHL18 acts as a tumor suppressor in NSCLC.

### BTB and non-BTB domains of KLHL18 are critical for the ubiquitination of PI3Kp85α

**Fig. 5:**
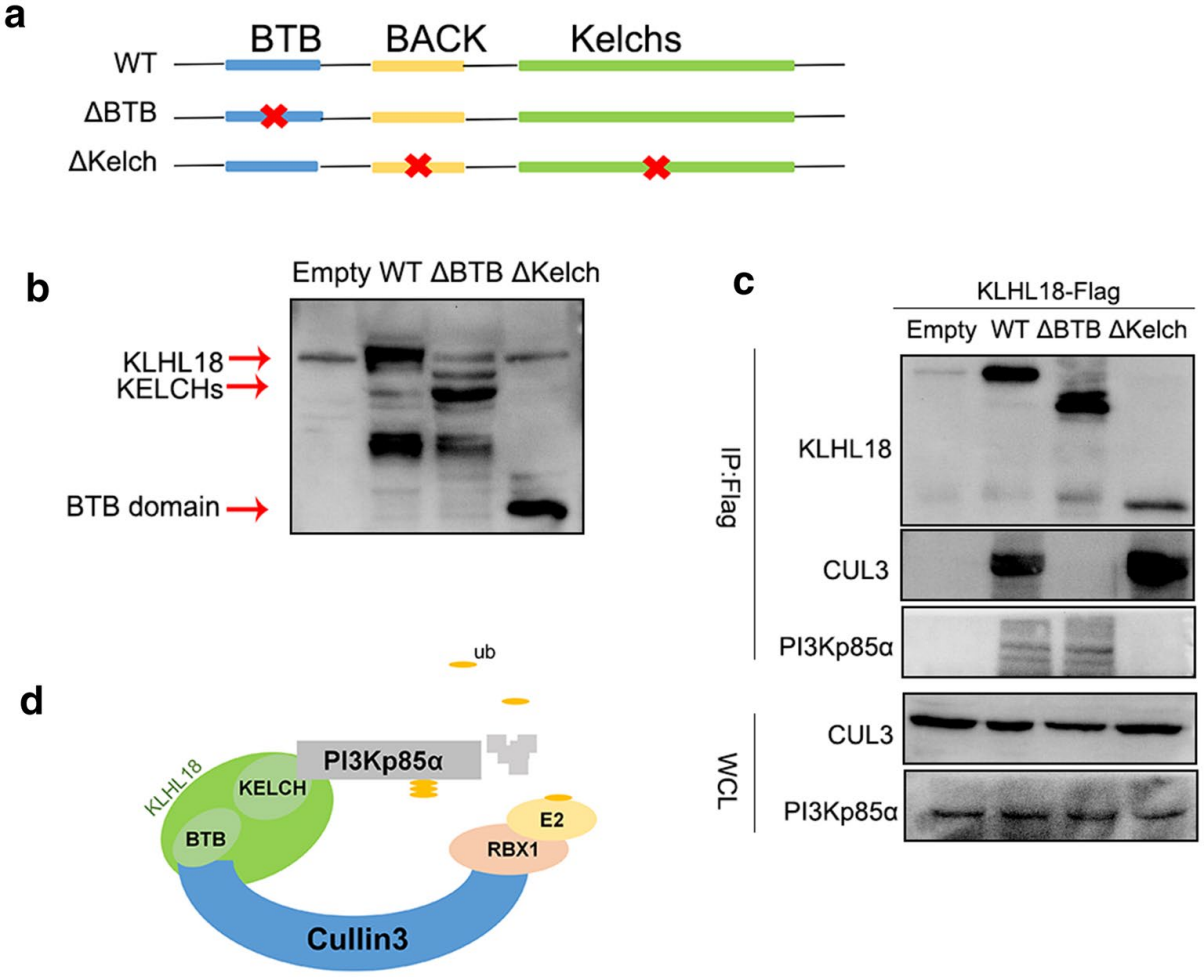
KLHL18 acts as an adaptor protein to interact with CUL3 and PI3Kp85α through different domains. **a** Locations of the deletions of KLHL18 domains. **b** Expression efficiency of KLHL18 splice variants in HEK-293 T cells. **c** Co-IP using the spliced variants of KLHL18 indicated that CUL3 bound to the BTB domain, whereas PI3Kp85α bound to the non-BTB domain. **d** The potential mechanism through which KLHL18 exerts its effects in NSCLC cell lines

To investigate which domain of KLHL18 is important for this function, we constructed a BTB domain-deleted splice variant (KLHL18-ΔBTB) and another with a fragment deleted between amino acids 140 and 571 (KLHL18-ΔKelch) (Fig. 5a). We then tested their effects on KLHL18-mediated ubiquitination of PI3Kp85α.

**Fig. 6:**
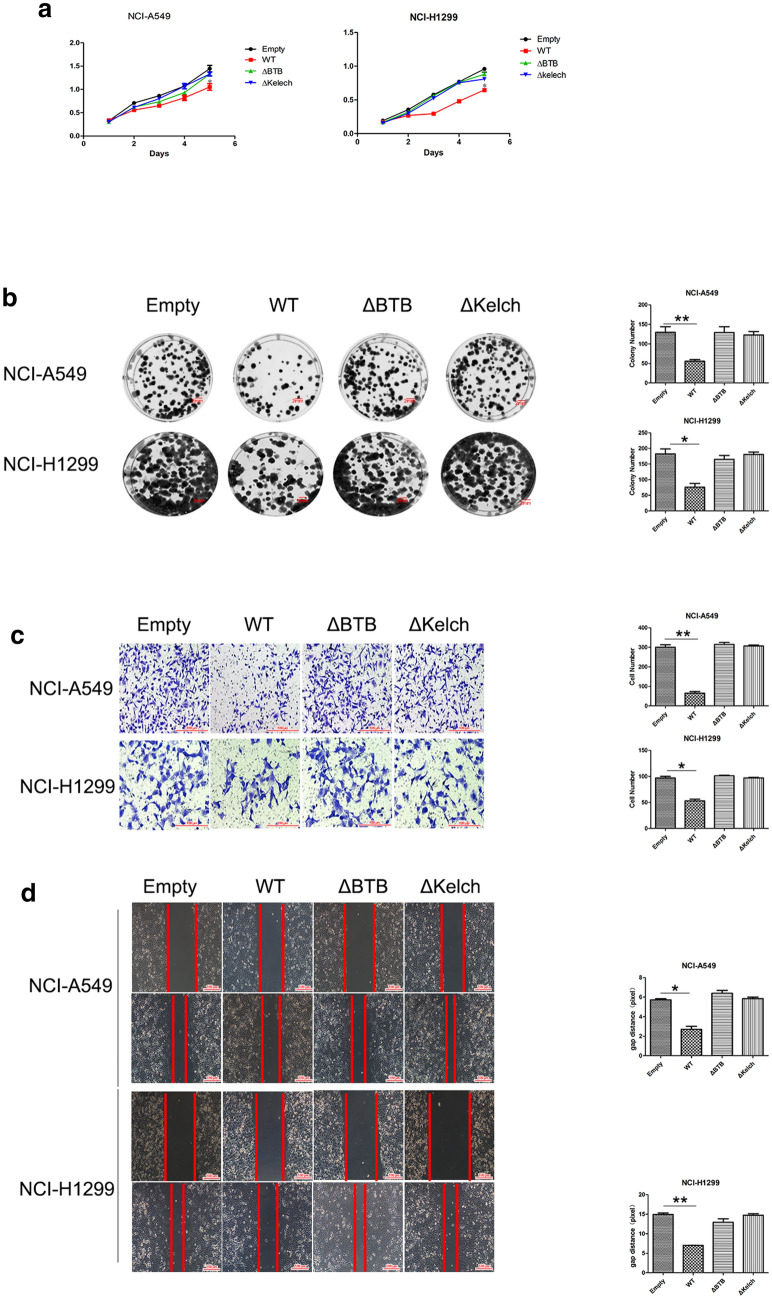
Role of BTB and non-BTB domains of KLHL18 in non-small cell lung cancer cells. **a** MTS assay based on KLHL18-ΔBTB- and KLHL18-ΔKelch-transfected NSCLC cell lines; left and right panels show NCI-A549 and NCI-H1299 cell lines, respectively, *P < 0.05, **P < 0.01. **b** The effect of KLHL18 and its splice variants on the colony-forming ability of NSCLC cell lines. The right panel shows the quantification of colonies,*P < 0.05, **P < 0.01. **c** The effect of KLHL18 and its splice variants on the invasive ability of NSCLC cells. The graph on the right side indicates the number of cells passing through the Matrigel, *P < 0.05, **P < 0.01. **d** The effect of KLHL18 and its splice variants on the migration of NSCLC cells. The graph on the right side indicates the cell migration distances *P < 0.05, **P < 0.01

**Fig. 7:**
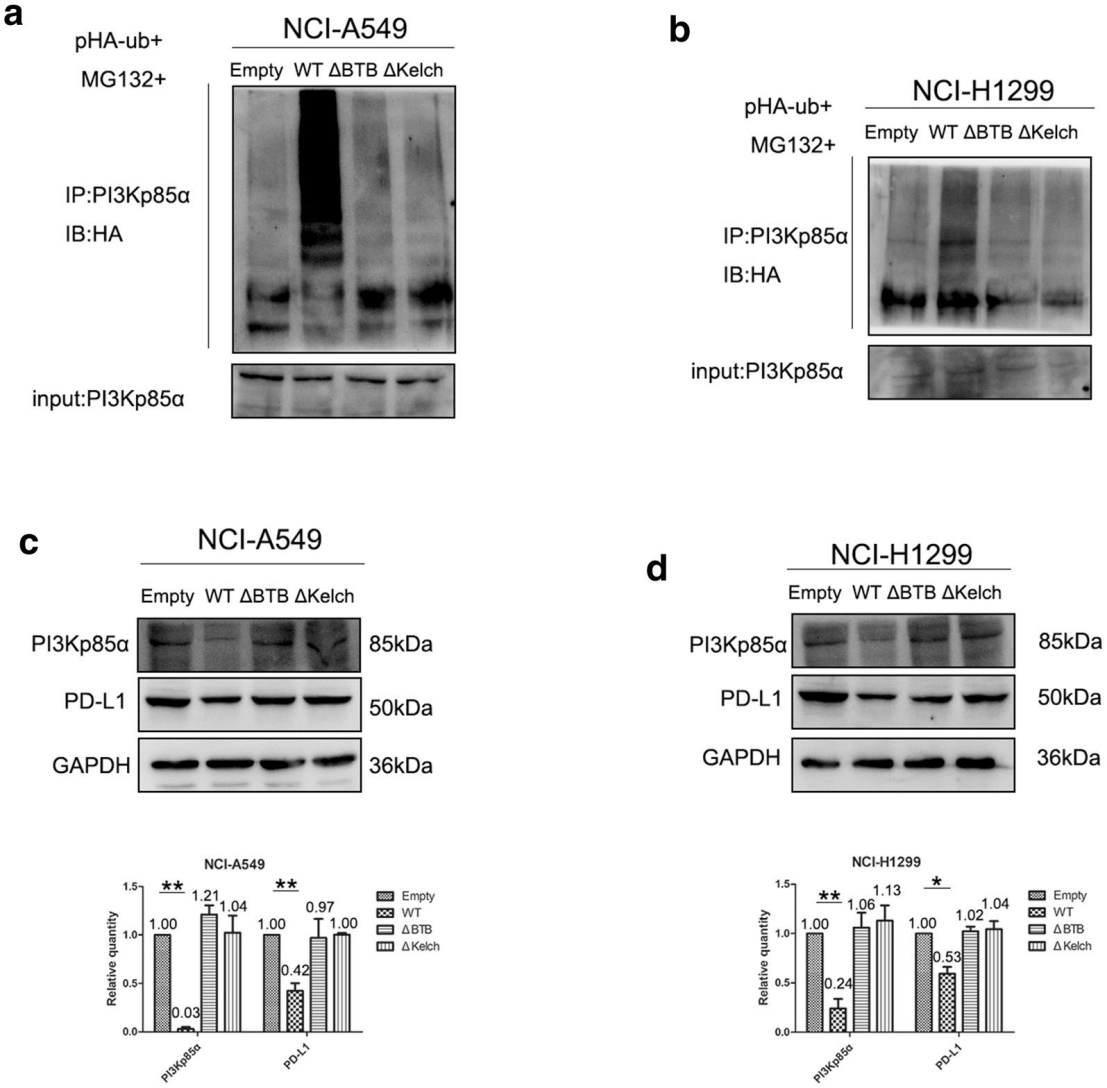
The BTB and non-BTB domains of KLHL18 are critical for PI3Kp85α ubiquitination and PD-L1 expression. **a** The effect of KLHL18 and its splice variants on the ubiquitination level of PI3Kp85α protein in NCI-A549 cells. **b** The effect of the KLHL18 splice variants on the ubiquitination level of PI3Kp85α protein in NCI-H1299 cells. **c**, **d** In NCI-A549 and NCI-H1299 cell lines, decreased PD-L1 protein levels were reversed with the deletion of the BTB domain or amino acids 140–571 near the C-terminus. The lower graph corresponds to the gray value statistical graph, *P < 0.05, **P < 0.01

First, the two splice variants were transfected into HEK-293 T (human embryonic kidney) cells, and the efficiency of expression was determined (Fig. 5b). Co-IP experiments were performed using two stably transfected HEK-293 T cell lines. KLHL18-ΔBTB did not interact with CUL3 protein (Fig. 5c). In these cells, KLHL18-Δkelch was unable to interact with PI3Kp85α (Fig. 5c). Immunofluorescence and Co-IPs also showed that PI3Kp85α binds to the non-BTB domain (Additional file 1: Figs. S4 and S5). This result confirms the hypothesis that the BTB domain and the amino acid fragment between positions 140 and 571 play a key role in the ubiquitination of PI3Kp85α (Fig. 5d). The proliferative ability of NCI-A549 cells was restored upon transfection with the two splice variants of KLHL18 (Fig. 6a), and the colony formation (Fig. 6b), cell invasion (Fig. 6c), and migration (Fig. 6d) abilities of these cells were stronger than those of cells transfected with KLHL18-WT. Similar results were observed for NCI-H1299 cells. Thus, the inhibitory effects of KLHL18 were eliminated by deleting its BTB domain or the fragment between amino acids 140 and 571. The ubiquitination of PI3Kp85α (Fig. 7a, b) and decreased levels of PD-L1 protein (Fig. 7c, d) were also suppressed by deleting the two fragments. These results indicate that the two domains participate in and promote the ubiquitination of PI3Kp85α, thereby inhibiting proliferation, migration, and invasion of NSCLC cells.

## Discussion

At present, the options for the early diagnosis of lung cancer are limited, and the mortality rate of lung cancer is among the highest for malignant tumors [28]. Both molecular therapy and immunotherapy offer great potential for the treatment of lung cancer [29]. Although the treatment process for lung cancer is relatively established, new methods and targets to improve and tailor treatment options are still necessary.

Our data show that the expression of KLHL18 is decreased in NSCLC with a decreasing degree of differentiation and is correlated with TNM stage, lymph node metastasis, and tumor size. Clinically, we found that subjects with high KLHL18 expression showed longer survival and had a better prognosis than those with low KLHL18 expression.

We also identified PI3Kp85α as the substrate of KLHL18, which binds PI3Kp85α and promotes its ubiquitination-mediated degradation. Ubiquitination is one of the important degradation pathways of proteins in vivo [30–33]. We found that KLHL18 inhibits the expression of the PD-L1 protein. Based on this discovery, we suspect that KLHL18 affects PD-L1 function by preventing its binding to its specific recognition protein PD-1, thereby inducing immune cells to kill tumor cells. Therefore, we theorize that the KLHL18 protein can regulate the immune microenvironment of tumor cells, thereby demonstrating its function as a tumor suppressor. However, this conjecture requires further research for its verification. This study only studied the potential mechanism of action of KLHL18 through in vitro experiments. Although additional in vivo experiments are warranted to test whether KLHL18 protein affects the sensitivity of tumor cells to immunosuppressive drugs, our findings have important implications for understanding the role and mechanism of action of KLHL18 in tumorigenesis.

## Conclusions

Our results indicate that KLHL18 can serve as a prognostic biomarker and potential immunotherapeutic target for NSCLC. The occurrence and development of tumors is a multifactorial process, and the KLHL18–PI3Kp85α–PD-L1 axis may have multiple functions in NSCLC pathogenesis. In this study, we investigated the clinical features of KLHL18 expression, performed survival analysis, and examined its effects in vitro, providing a foundation for considering KLHL18 as a new clinical therapeutic target.

## Methods

### Patients and specimens

From 2007 to 2017, 214 NSCLC tissue sections and 22 pairs of cancer and adjacent tissues were procured from the First Affiliated Hospital of China Medical University. This study was approved by the Ethics Committee of China Medical University, and all recruited patients provided informed consent. Before surgery, follow-up information of patients who were not administered radiotherapy or chemotherapy in the study was recorded in the patients’ medical records. The clinicopathological features of the cases are summarized in Table 1.

### NSCLC cell culture

All cell lines were purchased from the Shanghai Cell Bank (Shanghai, China) and cultured in medium supplemented with 10% fetal bovine serum (FBS; FB15015; Clark Biosciences, Richmond, VA, USA). HBE cells and HEK-293T cells were cultured in high-glucose Dulbecco’s modified Eagle’s medium; NCI-A549, NCI-H1299, NCI-H460, NCI-H661, and NCI-LK2 cells (NSCLC cell lines) were cultured in Roswell Park Memorial Institute 1640 medium; and SK-MES-1 cells (NSCLC squamous cell carcinoma cell line) were cultured in minimal essential medium.

### Plasmid construction and transfection

shKLHL18-GFP [sh279-191, RNA oligo sequences 5′-UGUCAUUUGUAAACAUAGCAU-3′] and scramble shRNA (negative control) were purchased from Sangon Biotech (Shanghai, China). KLHL18 (WT)-Flag plasmid (RC223003), and the control plasmid Pcmv6-Flag were purchased from Origene (Rockville, MD, USA). ΔBTB-Flag, containing a knockout of the amino acids from positions 28–131, and ΔKelch-Flag, containing a knockout of the amino acids from positions 140–571, are two splice variants of the KLHL18 full-length plasmid; these were obtained from TSINGKE Biological Technology (Beijing, China). Cell transfection was performed using Lipofectamine 3000 reagent (Invitrogen, Carlsbad, CA, USA) according to the manufacturer's instructions. KLHL18-shRNA and its negative control, as well as KLHL18-Flag plasmid and its control Pcmv6, were transfected into selected cell lines. At 72 h after transient transfection, G418 antibiotics (Thermo Fisher Scientific, Waltham, MA, USA) were added to screen the stably transfected cell lines, and monoclonal stably transfected cell lines were screened by the limiting dilution procedure to obtain stable clones. The cells were cultured in a 25 T cell culture flask.

### Immunohistochemistry

NSCLC tissue sections were incubated with anti-KLHL18 rabbit polyclonal antibody (17229-1-AP, 1:100 dilution; Protein Tech Group, Chicago, IL, USA) at 4 °C overnight, and the percentage of stained cells and staining intensity were calculated after color development [34].

### Western blotting

Total protein was extracted with an appropriate amount of lysis buffer (P0013; Beyotime Biosciences, Shanghai, China), and appropriate amounts of protease inhibitor and phosphatase inhibitor (B14002 and B15002, respectively; Biotool, Shanghai, China) were used to extract protein from 35 μg of samples. The ratio of protein lysate buffer, protease inhibitor, and phosphatase inhibitor was 100:1:1, and the total volume of the mixture was four times the cell volume. The following primary antibodies were used: anti-CUL3 (11107-1-AP, 1:100), anti-p27 (25614-1-AP, 1:500), anti-MMP9 (10375-2-AP, 1:1,000), and anti-FLAG (66008-2-Ig, 1:100) from Proteintech Group (Chicago, IL, USA); anti-phospho-mTOR (5536 s, 1:1,000), anti-mTOR (2983 s, 1:1,000), anti-PI3Kp85α (13666S, 1:1,000), anti-PI3Kp85α (4257S, 1:1,000), anti-AKT (4691S, 1:1,000), anti-phospho-AKT (4060, 1:1,000), anti-PD-L1 (13684S, 1:1,000), anti-RhoC (D40E4, 1:1,000), anti-N-Cadherin (13116 s, 1:1,000), anti-Snail (3879 s, 1:1,000), anti-Slug (9585 s, 1:1,000), and anti-HA-Tag (3724 s, 1:1,000) from Cell Signaling Technology (Danvers, MA, USA); and anti-GAPDH (TA319654, 1:1,000) from Origene. Proteins were visualized using enhanced chemiluminescence (34080; Thermo Fisher Scientific). ImageJ software (NIH, Bethesda, MD, USA) was used to quantitatively evaluate the grayscale integral value of each band [35].

### qPCR

SYBR Green PCR Master Mix was used for amplification in a 7900HT fast RT-PCR system (Applied Biosystems, Foster City, CA, USA) with a total volume of 20 μL. The reaction conditions were as follows: 95 °C for 30 s, followed by 40 cycles at 95 °C for 5 s, and 60 °C for 30 s. The dissociation step was used to generate a melting curve and confirm amplification specificity. Relative gene expression with *β-actin* as the reference gene was calculated using the 2^−ΔΔCt^ method. The sequences of the primers used were as follows: KLHL18 forward, 5′-AAGGCCTCTGCTTCTGAGAG-3′, and reverse, 5′-GATATCACACGGCATTCTGG-3′; β-actin forward, 5′-ATAGCACAGCCTGGATAGCAACGTAC-3′, and reverse, 5′-CACCTTCTACAATGAGCTGCGTGTG-3′.

### MTS proliferation assay

The cell lines stably transfected with the KLHL18 plasmids were seeded into a 96-well plate (3000 cells/well) and cultured for 5 days in a medium containing 10% FBS. Next, 20 μL of 3-(4,5-dimethylthiazol-2-yl)-5-(3-carboxymethoxyphenyl)-2-(4-sulfophenyl)-2H-tetrazole salt (MTS; G3580, Promega, Madison, WI, USA) was added to each well to test cell viability. After incubating the cells for 1 h at 37 °C in the dark, the color intensity of each plate was measured at 490 nm using a microplate reader.

### Colony formation assay

Cell lines stably transfected with KLHL18-related plasmids were seeded into six-well plates (800 cells/well) for colony formation assays and grown until they formed visible colonies (approximately 14 days). They were then washed three times with phosphate-buffered saline (PBS), fixed with pre-chilled methanol, washed three times with PBS, stained with crystal violet, air dried, and manually counted.

### Cell Transwell invasion analyses

Cell invasion assays were performed in a 24-well Transwell chamber containing an insert with a pore size of 8 μm (Costar, Washington, DC, USA). The insert was added to 100 μL Matrigel (1:9 dilution; BD Biosciences). Next, 5 × 10^4^ cells were added to 100 μL of 2% FBS, the mixture was added to the Transwell upper chamber, and 600 μL of 20% FBS was added to the lower chamber. After incubating the cells for 24 h in a sterile incubator at 37 °C, the upper surface cells were removed with a cotton swab and fixed with ice-cold methanol. Finally, the cells were stained with crystal violet staining solution, dried, and the number of cells invading the lower chamber was counted. The field of view was randomly selected under a microscope at a magnification of 20×.

### Wound-healing assays

The cells were grown in a single layer in a six-well plate until a growth density of approximately 90% was reached. The cells were incubated in a sterile incubator (1758-9327, Inalco, Beijing, China) in the dark at 37 °C for 2 h. Next, the cells were placed under sterile conditions, and the cell monolayer was scraped with a 100-μL pipette tip. After non-adherent cells were removed and washed with PBS, the cells were continuously cultured in serum for a prescribed period, and the treated cells were photographed under a microscope. The wound distance was quantitatively measured using ImageJ (NIH).

### Immunofluorescence

The cells were cultured in a special dish, imaged using confocal microscopy (801002, NEST, Hong Kong, China), and, after fixation with 4% paraformaldehyde/PBS for 15 min, treated with 0.1% Triton X-100 for 10 min. The cells were then washed three times with PBS, blocked with 3% bovine serum albumin (A8010, Solarbio, Beijing, China) for 2 h, and incubated with the primary antibody at 4 ℃ overnight. The next day, the cells were aspirated and washed with PBS three times, followed by incubation with a secondary antibody for 2 h at 24–27 °C. The cells were washed three times with PBS and stained with 4′,6-diamidino-2-phenylindole for 10 min. A confocal microscope (FV3000, Olympus, Tokyo, Japan) was used for imaging.

### Co-IP assays

The cell line of interest was plated in two 10-cm dishes. When 100% confluency was reached, the cells were lysed and centrifuged at 12,000 rpm for 15 min at 4 °C. The supernatant was collected for Co-IP, and 60 μL Protein A/G Sepharose (P2012; Beyotime Biosciences) was added to the supernatant to block for 2 h; the supernatant was then centrifuged at 1000 rpm for 5 min at 4 °C and divided into two parts on average; 4–10 μg of the target antibody and anti-mouse/rabbit IgG (1:2000; ZSGB-BIO, Beijing, China) were added, and the antibody was shaken overnight in a 4 °C chromatography cabinet. On the next day, 25 μL of agarose A/G magnetic beads were added to each tube and incubated at 4 °C for 6 h, after which the cell lysate was washed with lysis buffer, and the tubes were heated in boiling water for 10 min followed by immunoblotting.

### Ubiquitination assays and immunoprecipitation

The stably transfected cell line was transfected with the Ub-HA plasmid, and the 26S proteasome inhibitor MG-132 (HY-13259, MedChemExpress, Monmouth Junction, NJ, USA) was added at a final concentration of 18.5 μM. The assays were performed as described previously [36]. The immune complexes were collected by centrifugation, washed in cell lysis buffer, and subjected to immunoblot analysis.

### Mass spectrometry

Cells of interest were seeded into a 10-cm dish. After the cells reached confluence, they were collected, lysed for 20 min, and centrifuged at 12,000 rpm at 4 °C for 15 min. Next, 60 μL of Protein A/G Sepharose (P2012; Beyotime Biosciences) beads was added to the collected supernatant, followed by incubation for 2 h. The sample was then centrifuged at 1,000 rpm at 4 °C for 5 min, and the supernatant was divided evenly into two fresh tubes. Then, 4 μg of the indicated target antibody or the anti-mouse IgG (1:2,000; ZSGB-BIO) was incubated with the supernatant overnight at 4 °C. Next, 25 μL of agarose A/G magnetic beads was added to each sample, followed by incubation at 4 °C for 6 h. Samples were then washed 3 tims with the lysis buffer and then boiled for 10 min. Samples were then subjected to 10% SDS-PAGE. Gels were stained with the Coomassie brilliantblue (ST1119; Beyotime Biosciences) to stain the glue, choose the difference from the IgG group glue to add to the Q-Exactive mass spectrometer instrument (Thermo Scientific) for detection, MS/MS scanning range is 50–2200 m/z. The peak value is analyzed by Data Analysis Software, then perform a Moscot search to find matching proteins.

### Inhibitor treatments

MG-132 (HY-13259, MedChemExpress) is a proteasome inhibitor that can effectively prevent the hydrolysis of the 26S proteasome, which was added at a concentration of 18.5 μM for 24 h. MLN4924 (HY-70062, MedChemExpress) can selectively inhibit the activity of the NEDD8-activating enzyme, which is present at the C-terminus of CUL3 and can activate CRL by various modification pathways [36]; it was added at a concentration of 0.3 μM for 24 h. LY294002 (HY-10108, MedChemExpress) is a broad-spectrum inhibitor of PI3K, which was added at a concentration of 40 mM for 24 h. Rapamycin (HY-10219, MedChemExpress) is a specific mTOR inhibitor; it was added at a concentration of 15 nM for 12 h.

### Statistical analysis

All data were analyzed using SPSS version 24.0 (SPSS, Inc., Chicago, IL, USA) and GraphPad Prism 5 (GraphPad, Inc., La Jolla, CA, USA). The χ^2^ test was used to assess the correlation between clinicopathological factors and KLHL18 expression. All experiments were independently repeated at least three times under the same conditions. A value of *P* < 0.05 was considered statistically significant.

## Supplementary information

**Additional file 1: Figure S1.** MS/MS spectrum of PI3Kp85α protein related peptids. **Figure S2.** Changes in levels of EMT pathway-related proteins in NCI-A549 and NCI-H1299 cells. The lower graph is a gray value statistical graph, *P < 0.05, **P < 0.01. **Figure S3. A** and **B**. After adding LY294002 to NCI-A549 cells and NCI-H1299 cells, AKT phosphorylation and mTOR phosphorylation levels decreased. The lower graph is a gray value statistical graph, *P < 0.05, **P < 0.01. **C** and **D**. After adding rapamycin to NCI-A549 cells and NCI-H1299 cells, PD-L1 levels decreased. The lower graph is a gray value statistical graph, *P < 0.05, **P < 0.01. **Figure S4.** A. Cellular immunofluorescence shows that PI3Kp85α does not bind to the BTB domain. B. Cellular immunofluorescence shows that PI3Kp85α binds to the non-BTB domains. **Figure S5. A** and **B**. As demonstrated via Co-IPs in NCI-A549 cells and NCI-H1299 cells, KLHL18-∆BTB can still bind to PI3Kp85α protein.

## Data Availability

All data generated or analyzed during this study are included in this published article. Further details are available from the corresponding author upon request.

## Abbreviations

Co-IP: Co-immunoprecipitation
CRL: Cullin-RING ligase
CUL3: Cullin 3
FBS: Fetal bovine serum
KLHL18: Kelch-like protein 18
NSCLC: Non-small cell lung cancer
PBS: Phosphate-buffered saline
PI3K: Phosphatidylinositol 3-kinase
qPCR: Quantitative polymerase chain reaction
LUAD: Lung adenocarcinoma
LUSC: Lung squamous cell carcinoma
