# Supplementary material for: KLHL18 inhibits the proliferation, migration, and invasion of non-small cell lung cancer by inhibiting PI3K/PD-L1 axis activity
Source: Cell Biosci. 2020 Nov 27;10:139. doi: 10.1186/s13578-020-00499-9 (PMC7694932; doi:10.1186/s13578-020-00499-9)
Supplement: Supplementary file 1 — Additional file 1: Figure S1. MS/MS spectrum of PI3Kp85α protein related peptids. Figure S2. Changes in levels of EMT pathway-related proteins in NCI-A549 and NCI-H1299 cells. The lower graph is a gray value statistical graph, *P < 0.05, **P < 0.01. Figure S3. A and B. After adding LY294002 to NCI-A549 cells and NCI-H1299 cells, AKT phosphorylation and mTOR phosphorylation levels decreased. The lower graph is a gray value statistical graph, *P < 0.05, **P < 0.01. C and D. After adding rapamycin to NCI-A549 cells and NCI-H1299 cells, PD-L1 levels decreased. The lower graph is a gray value statistical graph, *P < 0.05, **P < 0.01. Figure S4. A. Cellular immunofluorescence shows that PI3Kp85α does not bind to the BTB domain. B. Cellular immunofluorescence shows that PI3Kp85α binds to the non-BTB domains. Figure S5. A and B. As demonstrated via Co-IPs in NCI-A549 cells and NCI-H1299 cells, KLHL18-∆BTB can still bind to PI3Kp85α protein. [file 13578_2020_499_MOESM1_ESM.docx]

**Additional file 1: Figs. S1–S5.**


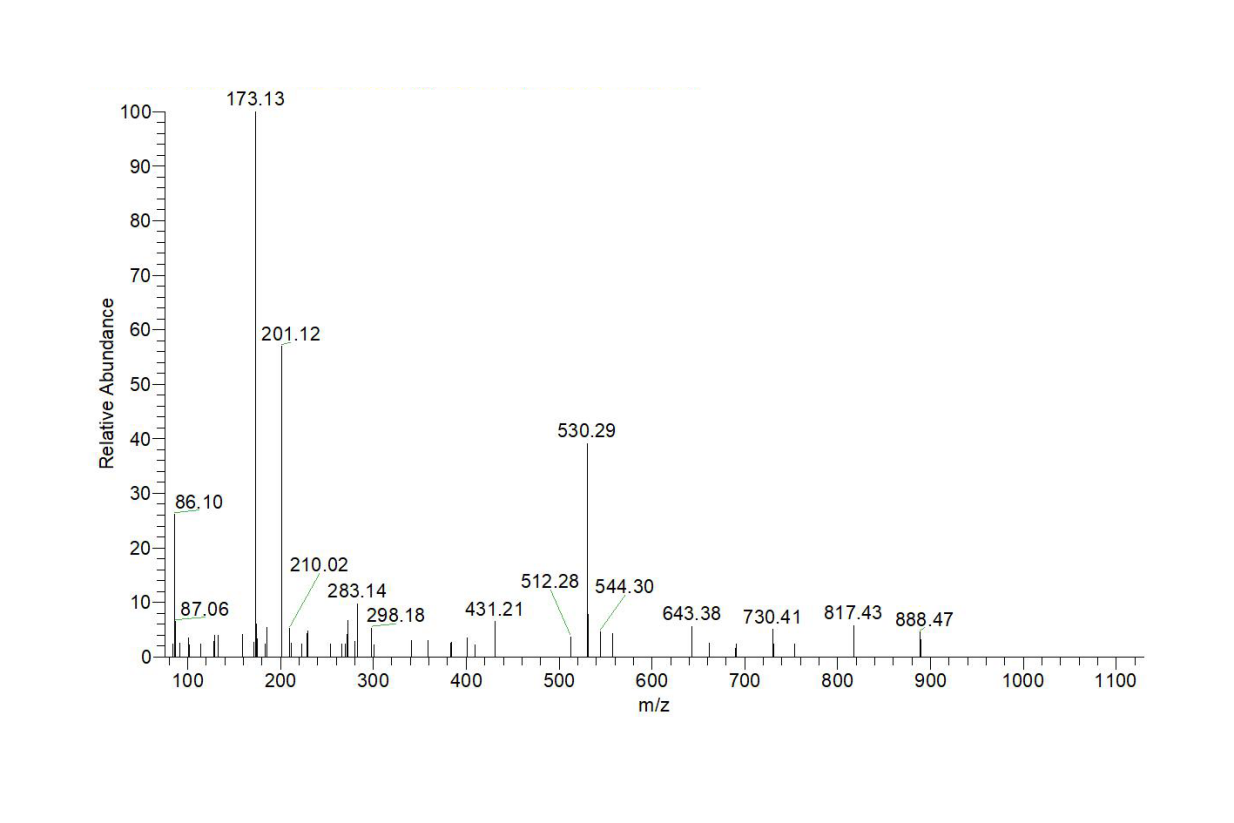


**Fig. S1. Mass spectrometry results**

MS/MS spectrum of PI3Kp85α protein related peptids.


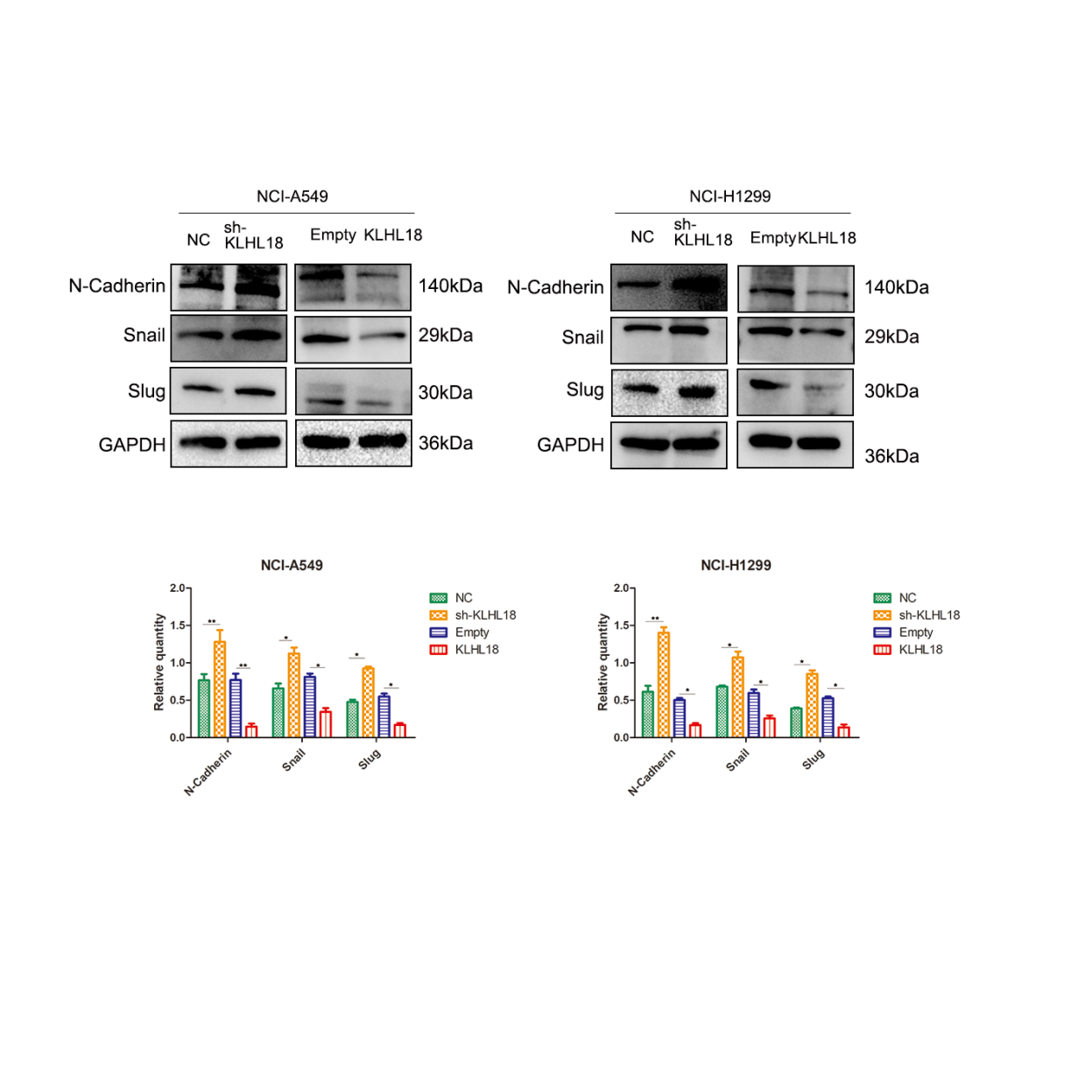


**Fig. S2. KLHL18 can regulate the expression of EMT pathway related proteins**

Changes in levels of EMT pathway-related proteins in NCI-A549 and NCI-H1299 cells. The lower graph is a gray value statistical graph, *P < 0.05, **P < 0.01.


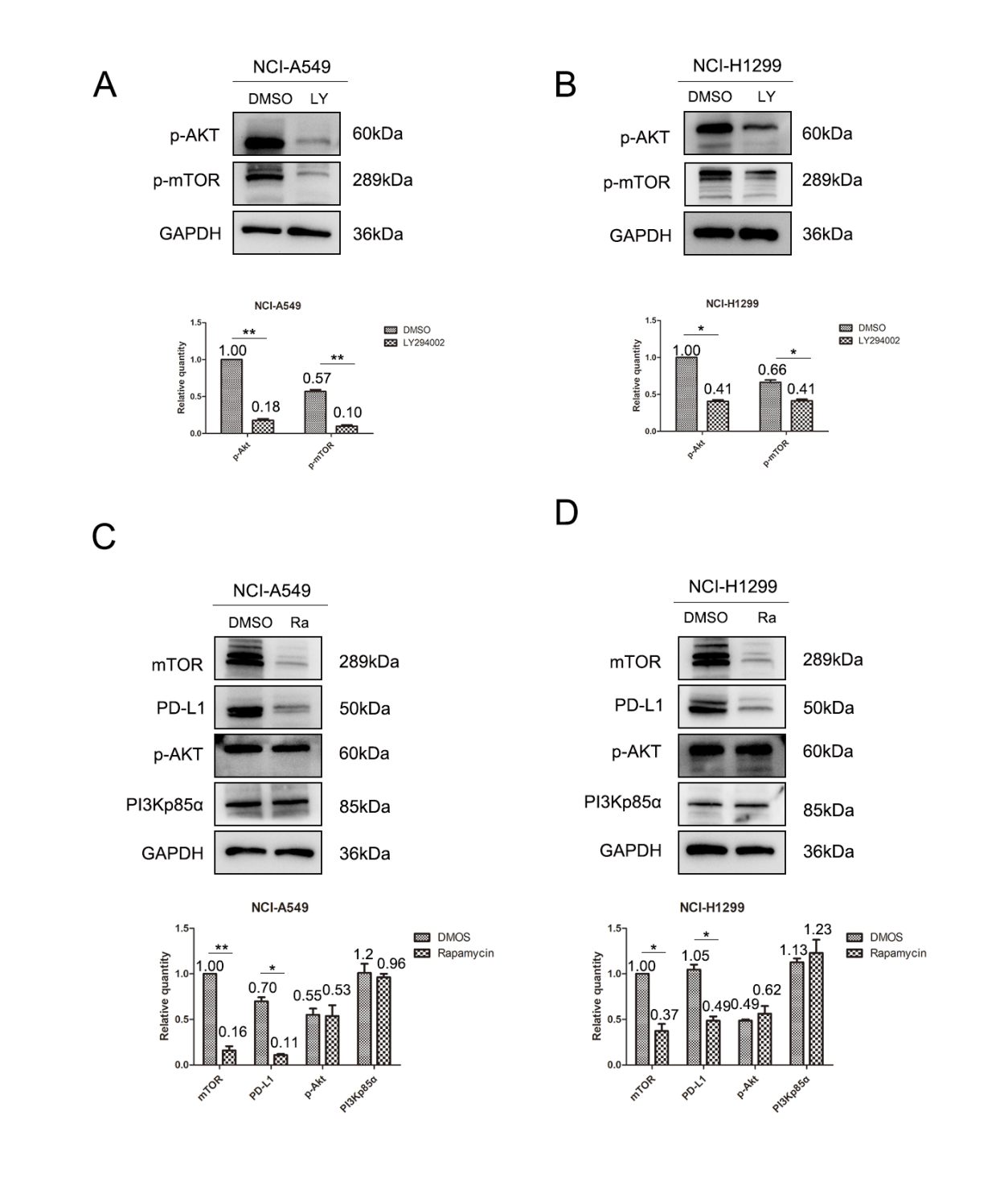


**Fig. S3. PI3K signaling pathway can regulate the expression of mTOR**

**A** and **B**. After adding LY294002 to NCI-A549 cells and NCI-H1299 cells, AKT phosphorylation and mTOR phosphorylation levels decreased. The lower graph is a gray value statistical graph, *P < 0.05, **P < 0.01. **C** and **D**. After adding rapamycin to NCI-A549 cells and NCI-H1299 cells, PD-L1 levels decreased. The lower graph is a gray value statistical graph, *P < 0.05, **P < 0.01.


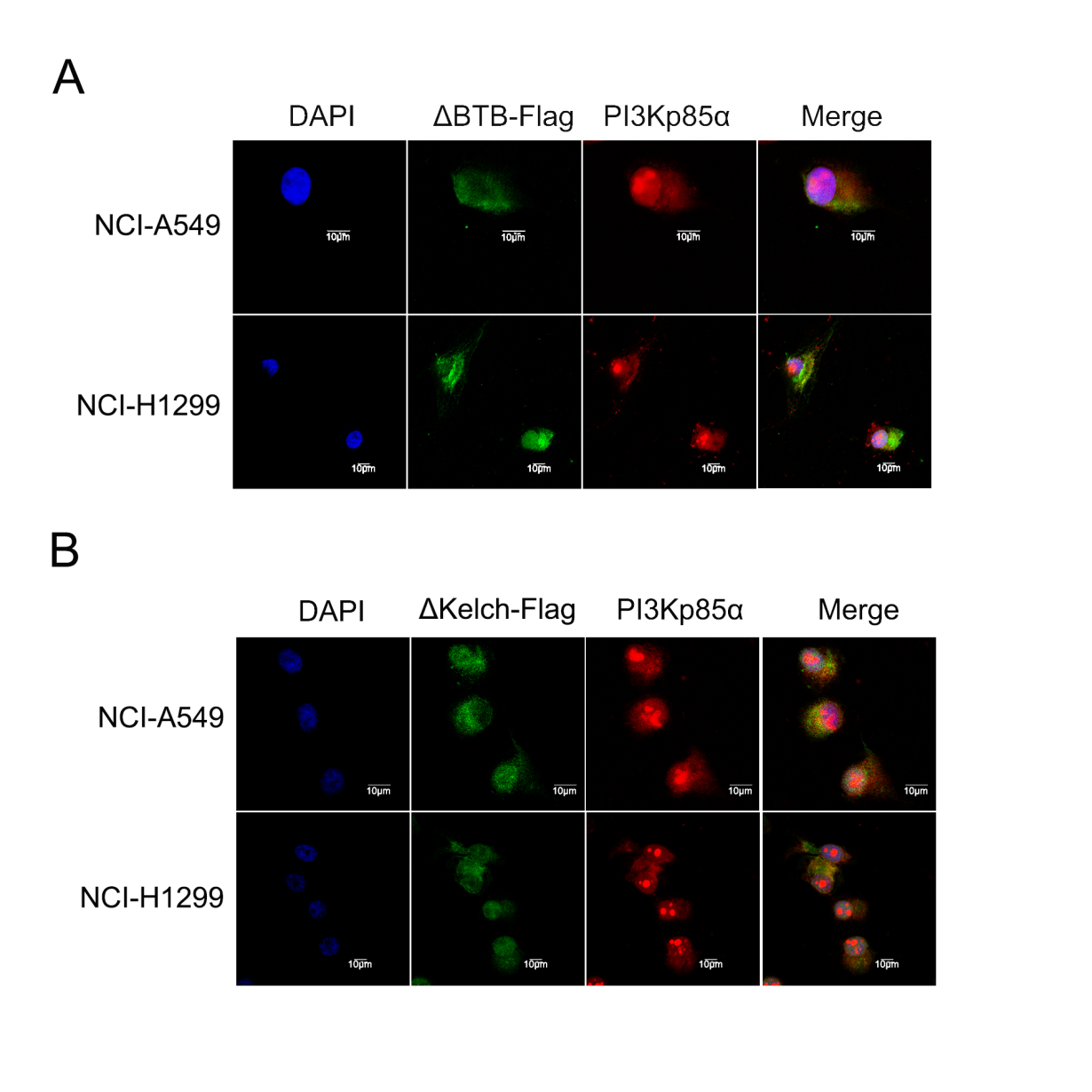


**Fig. S4. The immunofluorescence experiment of KLHL18 spliceosome**

1. Cellular immunofluorescence shows that PI3Kp85α does not bind to the BTB domain.
2. Cellular immunofluorescence shows that PI3Kp85α binds to the non-BTB domains.


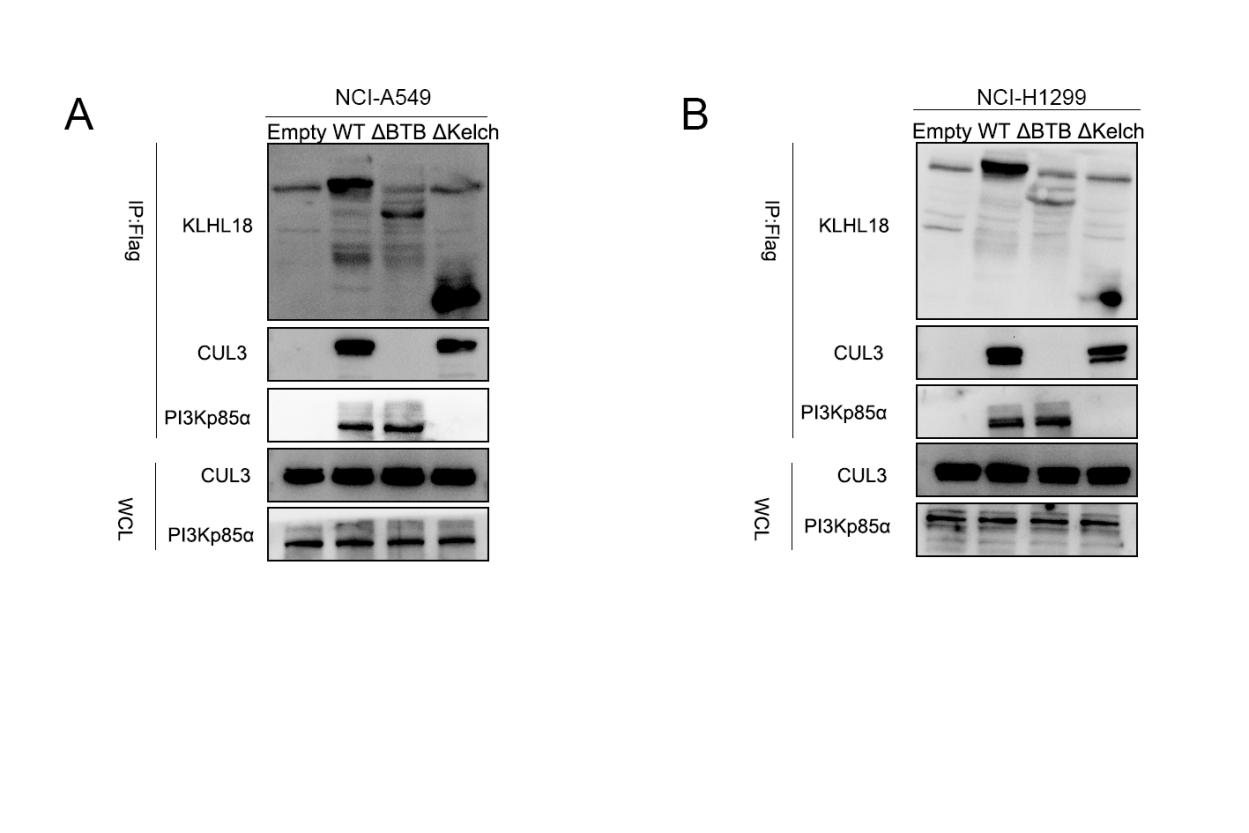


**Fig. S5. The KLHL18 spliceosome still has the ability to form complexes**

**A** and **B**. As demonstrated via Co-IPs in NCI-A549 cells and NCI-H1299 cells, KLHL18-ΔBTB can still bind to PI3Kp85α protein.
